# The benefit of bevacizumab therapy in patients with refractory vasogenic edema caused by brain metastasis from lung and colon cancers

**DOI:** 10.3389/fonc.2022.838670

**Published:** 2022-09-29

**Authors:** Xuexue Bai, Meng Zhou

**Affiliations:** Neurosurgery of The First Affiliated Hospital, Jinan University, Guangzhou, China

**Keywords:** bevacizumab, refractory brain edema, lung cancer, colon cancer, brain metastasis

## Abstract

**Objective:**

This retrospective study investigated the efficacy of bevacizumab in refractory brain edema caused by brain metastasis from lung cancer and colon cancer.

**Methods:**

A total of 72 patients with refractory brain edema were divided into the lung cancer and colon cancer groups according to their primary tumor. All patients received a single bevacizumab treatment for refractory brain edema. MRI was performed 1 week before the treatment and 4 weeks after the treatment. The edema and tumor volumes were calculated using imaging modalities.

**Results:**

After a single bevacizumab treatment, the refractory brain edema of 61 patients was controlled, and the clinical symptoms of 65 patients were improved. The average edema volume before treatment was 201,708.97 ± 61,426.04 mm^3^, which has decreased to 116,947.01 ± 43,879.16 mm^3^ after treatment (P < 0.05). After treatment, the edema index decreased from 25.97 ± 7.15 to 17.32 ± 5.24 (P < 0.05).We found that brain edema was controlled in 40 patients (93.02%) in the lung cancer group and 21 patients (72.41%) in the colon cancer group (P<0.05). In addition, 22 patients (88.00%) in the radiotherapy group achieved edema control, compared to 39 (82.98%) in the non-radiotherapy group (P>0.05). Nine patients experienced hypertension after treatment, two patients exhibited decreased platelet counts, and no hemorrhage cases were observed.

**Conclusion:**

Bevacizumab can significantly alleviate refractory brain edema, and there is a significant difference in the efficacy of bevacizumab on refractory brain edema caused by brain metastasis from lung and colon cancers.

## Introduction

Brain metastases are 10 times more common than primary intracranial cancer and represent the most common intracranial malignancy in adults (1, 2). Brain edema often occurs around brain metastases due to the abnormal accumulation of fluid in the brain parenchyma (3), which increases brain volume and elevated intracranial pressure (ICP) within the skull (4). Elevated ICP may decrease cerebral blood flow, causing hypoxia in the brain tissue and even brain herniation. These factors can lead to irreversible damage to nerve function and even death. Mannitol, diuretics, and steroids are used to reduce brain edema, but their therapeutic effect on refractory brain edema is unsatisfactory. Previous studies have shown that the control rate of these drugs for refractory brain edema is 27%–39% (5–10). These drugs cannot eliminate potential pathogenic factors and have many adverse reactions (11). The long-term use of steroids can lead to significant systemic side effects, including immunosuppression and avascular necrosis (12, 13). Mannitol may cause systemic hypotension, decreased cerebral perfusion, and acute renal failure (14, 15). Vascular endothelial growth factor A (VEGF-A) promotes angiogenesis and vascular permeability (16). Therefore, it is considered to play a key role in brain tumor-related edema. Bevacizumab, a monoclonal antibody against VEGF-A, is an effective treatment for brain edema (17–20). The purpose of this study is to explore whether there is a difference in the efficacy of bevacizumab for refractory brain edema caused by brain metastasis from lung and colon cancers.

We divided 72 patients who met the inclusion criteria into a lung cancer group (n=43) and colon cancer group (n=29) according to their primary tumor site of origin. We demonstrated that bevacizumab is effective for the treatment of refractory cerebral edema. Furthermore, the efficacy of bevacizumab for the treatment of refractory cerebral edema caused by metastatic tumors with distinct anatomical origins is different.

## Materials and methods

### Patients

From January, 2014 to January, 2021, 287 patients were treated with bevacizumab in our hospital. The inclusion criteria were as follows: (1) peritumoral brain edema confirmed by magnetic resonance imaging (MRI) examination; (2) clinical symptoms were not improved after more than 5 days of mannitol or glucocorticoid treatment; and (3) patients underwent pathological testing. The exclusion criteria were as follows: (1) patients with a history of hypertension; (2) patients with a history of other tumors; (3) patients with incomplete clinical data; and (4) patients who refused to sign the informed consent. All patients signed a written informed consent form before receiving bevacizumab treatment. The academic and ethics committee of the First Affiliated Hospital of Jinan University approved this study.

### Demographic characteristics

**Table 1** summarizes the demographic characteristics of the enrolled patients. A total of 72 patients were divided into a lung cancer group (n=43) and colon cancer group (n=29) according to the source of the primary tumor. There were 39 male patients and 33 female patients in this study. The average age was 61.75 ± 12.60 (range, 29–87 years). Of the 72 patients, 64 were diagnosed with brain metastases for the first time and had not received any treatment. Eight patients experienced tumor recurrence after craniotomy. None of the patients had a history of radiation therapy prior to bevacizumab treatment. In total, 25 patients received radiotherapy during MRI examination.

**Table 1:** Demographic characteristics of two groups.

	Lung cancer	Colon cancer	P
Age (Y)	62.51 ± 12.31	60.62 ± 13.15	>0.05
Sex			>0.05
Male	23	16	
Female	20	13	
KPS	62.79 ± 9.84	60.00 ± 12.82	>0.05
Tumor size (mm)	8.95 ± 3.11	8.41 ± 3.26	>0.05
Edema volume	201,558.70 ± 59,327.27	201,931.79 ± 65,482.65	>0.05
Edema index	25.60 ± 7.47	26.52 ± 6.73	>0.05
Treatment time	1	1	>0.05
History of craniotomy	5	3	>0.05
Radiotherapy (mean ± SE)			>0.05
Stereotactic radiotherapy	8(13.56 ± 2.53 Gy)	5(14.72 ± 1.24 Gy)	
Whole-brain radiotherapy	5(16.31 ± 4.25 Gy)	4(17.76 ± 3.28 Gy)	
Intensity-modulated radiotherapy	1(20Gy)	2(20.50 ± 0.71 Gy)	

### Treatment

Previous studies have suggested that the therapeutic dose of bevacizumab was 5 or 10 mg/kg (21, 22). The relationship between the bevacizumab dose and adverse reactions is unclear (23, 24). The purpose of utilizing bevacizumab in this study was to control refractory brain edema, so the therapeutic dose we used was 5 mg/kg. All patients received a single dose of bevacizumab.

### Imaging examination

**Figure 1:**
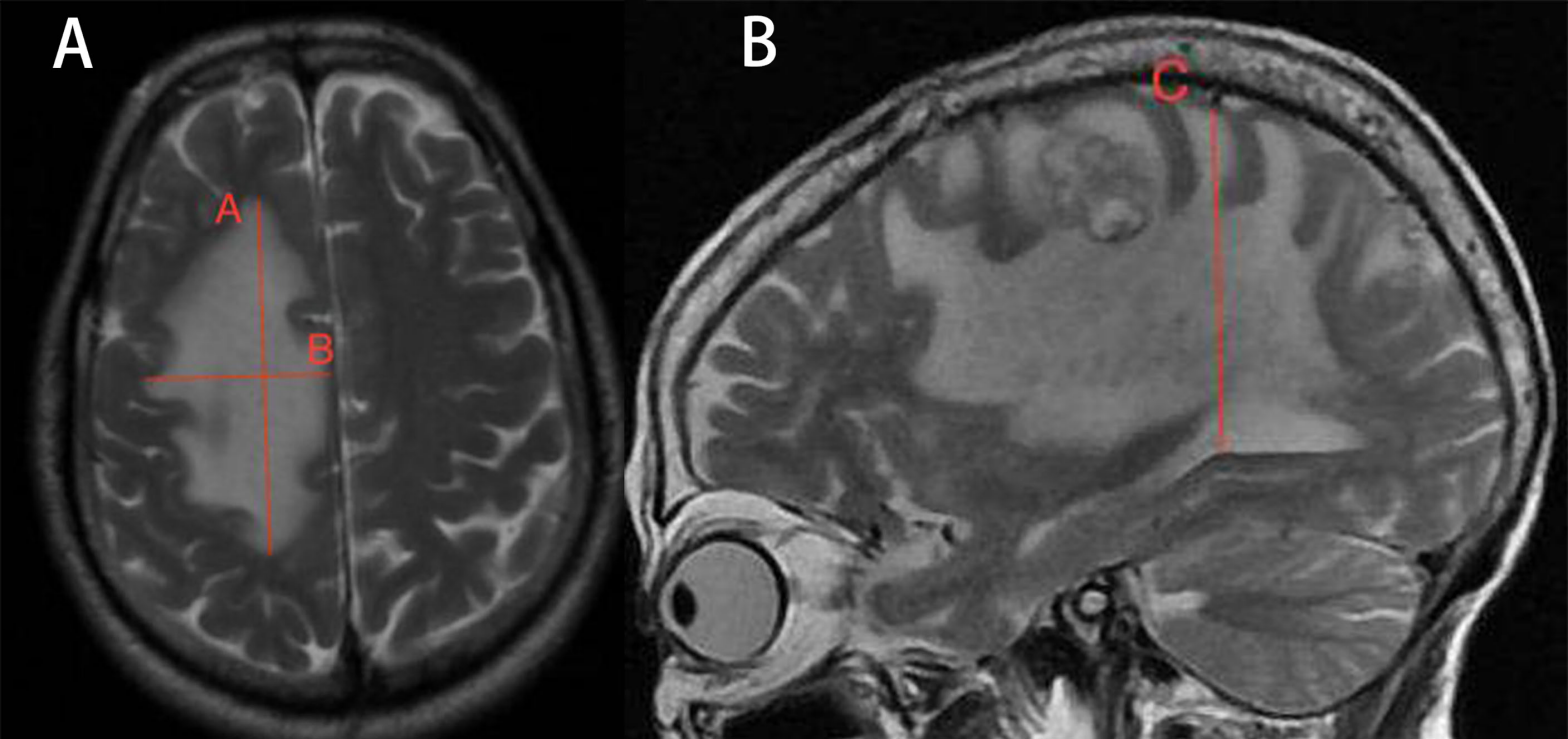
Demonstration of volume calculation technology. Volume calculation formula: V = π/6 × ABC. Volume is measured by drawing the mutually perpendicular diameters **(A, B)** of the largest cross-section of cerebral edema in the axial plane and maximum height of sagittal cerebral edema **(C)**. These measurements are substituted into the formula above to complete the volume calculation.

MRI was performed 1 week before the treatment and 4 weeks after treatment (25, 26). The tumor volumes were measured using T1-weighted images, and edema volumes were calculated using FRFSE and T2-weighted images. The tumor and edema volumes were measured using a method previously described by Bitzer (27). It is assumed that the volume of the tumor and brain edema is an elliptical sphere. Therefore, V = π/6 × ABC calculates the volume. **Figure 1** demonstrates the volume measurement technique. Volume is measured by drawing mutually perpendicular diameters (A and B) of the largest cross-section of cerebral edema in the axial plane and maximum height of sagittal cerebral edema (C). These measurements are substituted into the formula above to complete the volume calculation. The edema index (EI) was calculated as (volume of edema + tumor volume)/tumor volume (27). Edema volume reduction >10% was considered controlled, and volume increase or change ≤10% was considered uncontrolled (28). A total of 25 patients received radiotherapy during MRI examination. In total, 13 patients received stereotactic radiotherapy. Nine patients received whole-brain radiotherapy, and three patients received intensity-modulated radiotherapy.

### Statistical analyses

Our data were analyzed using SPSS 26.0 statistical software. The Wilcoxon signed-rank test was used to compare the differences in the edema volume and EI before and after bevacizumab treatment. The edema control rate of each group was compared using the chi-square test. An arbitrary level of 5% was used to indicate statistical significance.

## Results

### Therapeutic effect

**Figure 2:**
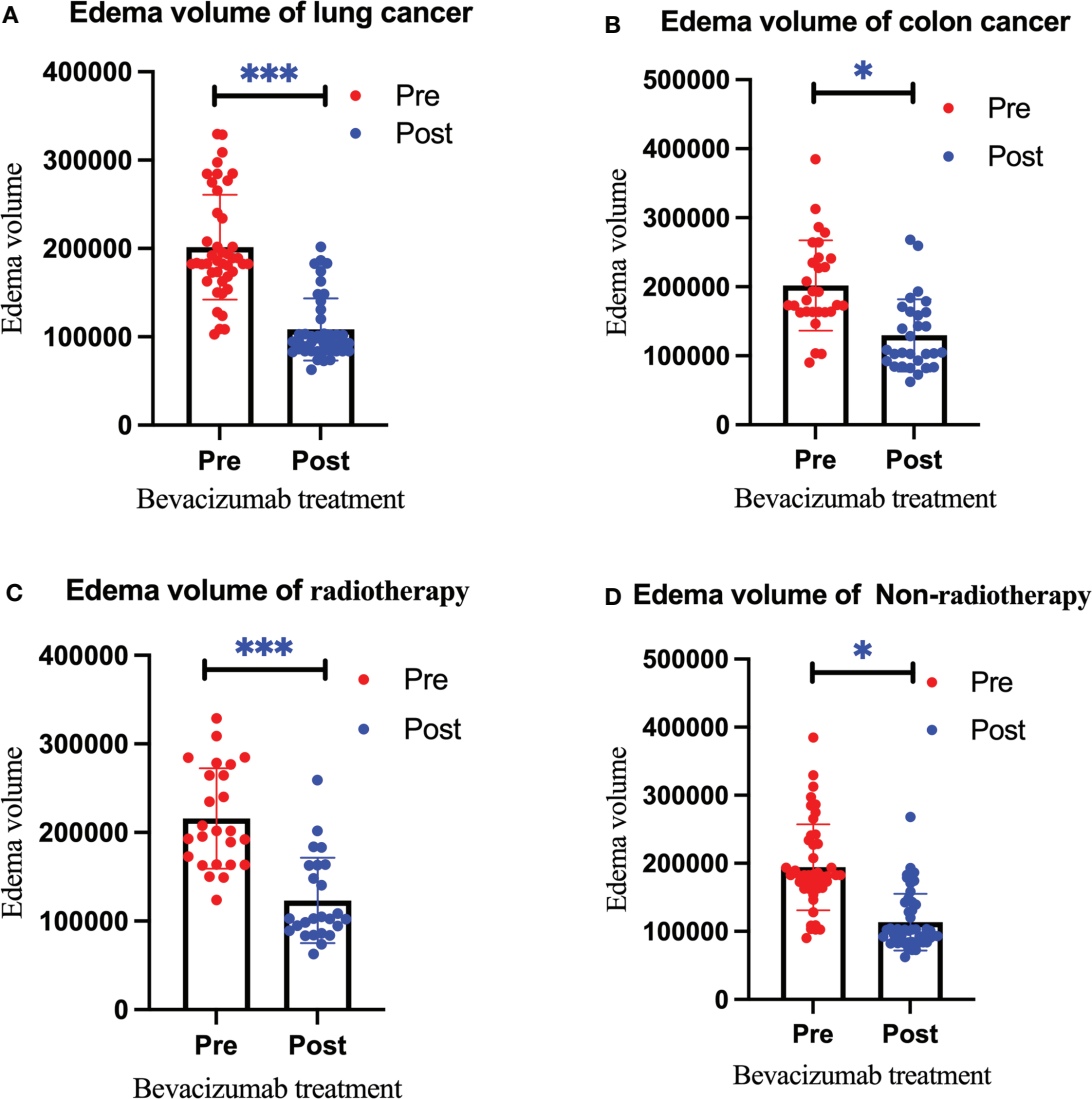
Changes in the edema volume before and after bevacizumab treatment in the lung cancer group **(A)**, colon cancer group **(B)**, radiotherapy group **(C)**, and non-radiotherapy group **(D)**. Red represents the edema volume before treatment, and blue represents the edema volume after treatment. We use * to indicate statistical difference. * for p<0.05, and *** for p<0.001.

**Figure 3:**
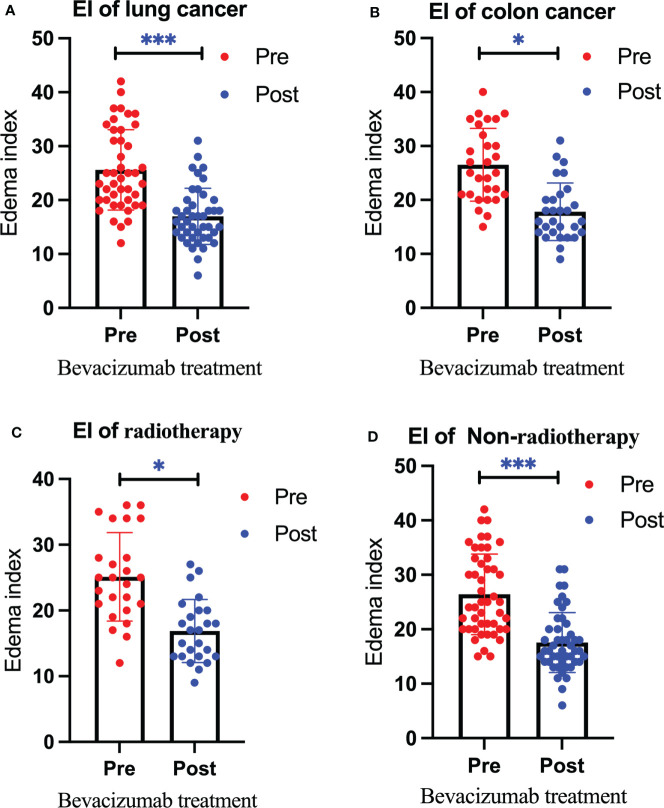
Changes in the edema index before and after bevacizumab treatment in the lung cancer group **(A)**, colon cancer group **(B)**, radiotherapy group **(C)**, and non-radiotherapy group **(D)**. Red represents the edema index before treatment, and blue represents the edema index after treatment. We use * to denote statistical differences. * for p<0.05, and *** for p<0.001.

**Figure 4:**
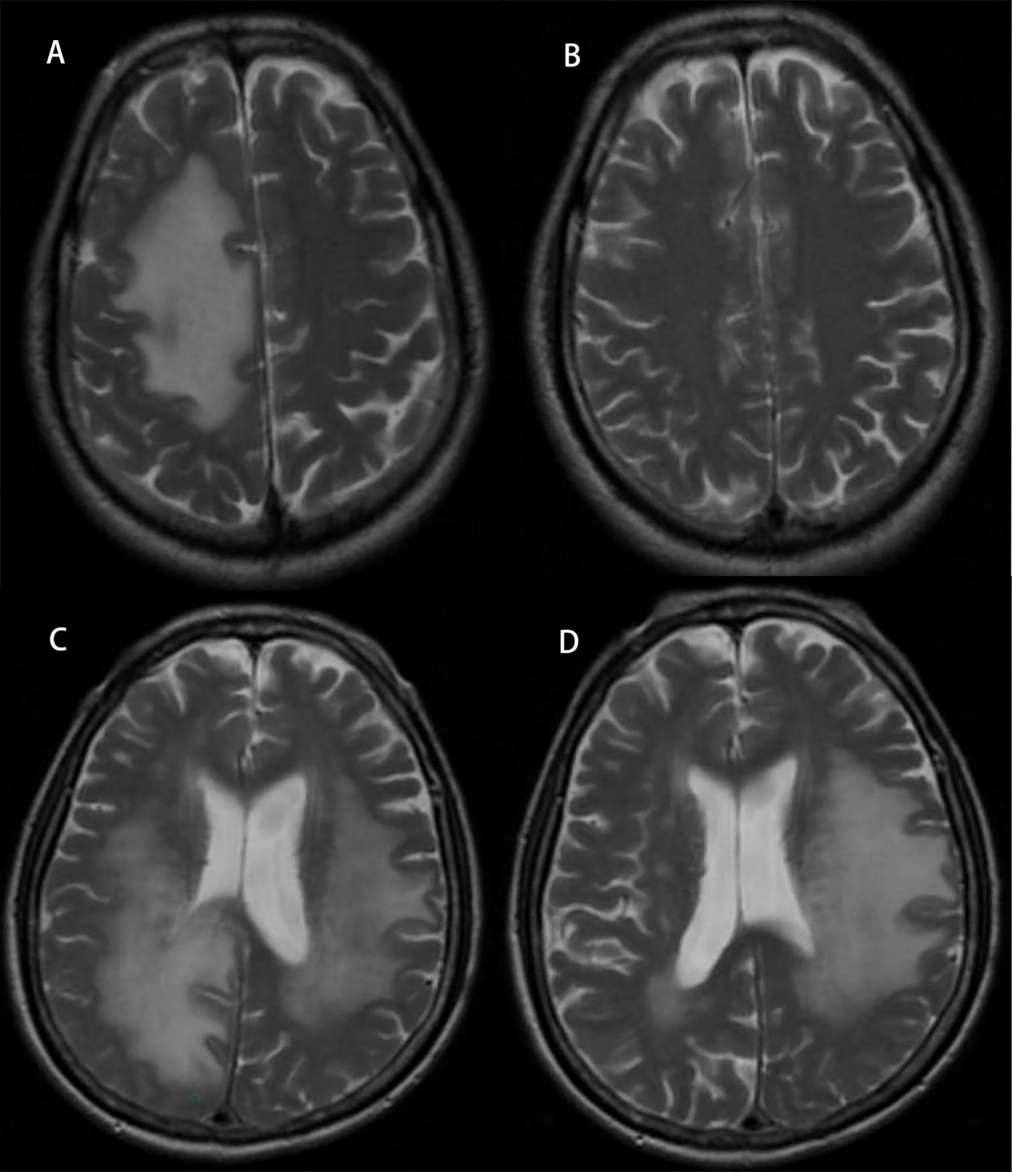
Radiographic images of brain edema before and after treatment with bevacizumab. Panel **(A)** represents edema in a lung cancer patient before treatment, and panel **(B)** represents after treatment. Panel **(C)** represents edema in a colon cancer patient before treatment, and panel **(D)** represents after treatment.

After treatment, the edema control rate was calculated by examining images. The results revealed that the refractory brain edema was controlled in 61 patients and the clinical symptoms were improved in 65 patients. **Table 2** summarizes the changes in the edema volume and EI before and after treatment in each group. **Figures 2**, **3** describe the changes in edema volume and EI before and after treatment, respectively. The results showed that bevacizumab effectively treated refractory brain edema and reduced EI. **Figure 4** shows the imaging changes in patients with lung cancer and colon cancer before and after treatment. **Table 3** compares the edema control rate in each group after treatment.

**Table 2:** Changes in edema volume and edema index after treatment.

	Pretreatment (x ± s)	Posttreatment (x ± s)	P
All (n=72)			
Edema volume (mm^3^)	201,708.97 ± 61,426.04	116,947.01 ± 43,879.16	<0.05
Edema index	25.97 ± 7.15	17.32 ± 5.24	<0.05
Lung cancer (n=43)			
Edema volume (mm^3^)	201,558.70 ± 59,327.27	108,344.40 ± 35,299.96	<0.001
Edema index	25.60 ± 7.47	16.98 ± 5.21	<0.001
Colon cancer (n=29)			
Edema volume (mm^3^)	201,931.79 ± 65,482.65	129,702.62 ± 52,258.17	<0.05
Edema index	26.52 ± 6.73	17.83 ± 5.34	<0.05
Radiotherapy (n=25)			
Edema volume (mm^3^)	215,883.08 ± 56,569.51	123,312.40 ± 48,058.32	<0.001
Edema index	25.12 ± 6.73	16.88 ± 4.79	<0.05
Non-radiotherapy (n=47)			
Edema volume (mm^3^)	194,169.55 ± 63,141.89	113,561.17 ± 41,629.79	<0.05
Edema index	26.43 ± 7.39	17.55 ± 5.50	<0.001

**Table 3:** Edema control rate of each group after treatment.

	Controlled n/N (%)	Uncontrolled n/N (%)	P
All (n=72)	61/72 (84.72%)	11/72 (15.28%)	
Lung cancer (n=43)	40/43 (93.02%)	3/43 (6.98%)	
Colon cancer (n=29)	21/29 (72.41%)	8/29 (27.59%)	
			<0.05
Radiotherapy (n=25)	22/25 (88.00%)	3/25 (12.00%)	
Non-radiotherapy (n=47)	39/47 (82.98%)	8/47 (17.02%)	
			>0.05

### Adverse reactions

Adverse reactions to bevacizumab included hypertension, several types of bleeding, venous thrombus exfoliation, and albuminuria (29, 30). The correlation between the drug dose and adverse reactions is unclear. Besse reported that the incidence of intracerebral hemorrhage in patients with brain metastases was 0.8%–3.3% after bevacizumab, while the incidence without bevacizumab was 1.0% (31). Khasraw reported that the incidence of intracerebral hemorrhage in patients with glioma or brain metastasis after bevacizumab treatment was 3.7%, while the incidence in those not administered bevacizumab was 3.6% (32). In addition, other complications after bevacizumab treatment have been reported, such as thrombocytopenia, intestinal perforation, and sepsis (33). In our study, nine patients experienced hypertension after treatment, two patients exhibited decreased platelet counts, and no cases of hemorrhage were observed.

## Discussion

Surgery is often considered first-line treatment for patients with a large (usually defined as >3 cm in diameter) or symptomatic brain metastasis; however, many patients are not optimal candidates for resection due to medical comorbidities, extensive extracranial burden of disease, or multiple intracranial metastases (34). None of the patients in this study were able to undergo craniotomy for various reasons. In these cases, radiation, either as whole-brain radiotherapy or stereotactic radiosurgery, is considered. There is a protracted response time following radiotherapy, with the earliest reaction observed within 2–3 months (35). Because the onset of radiation therapy was longer than our follow-up period, we believe that the effects of radiation therapy on cerebral edema in the patients during this study were small. Furthermore, although there is no definitive time limit, radiation-associated cerebral edema usually appears 3 or more months after radiation therapy (5).

Steroids are widely used to control clinical symptoms caused by perifocal edema (36). However, steroid treatment has side effects that impair the quality of life, including iatrogenic Cushing syndrome, which is frequently evident after only a few weeks of treatment (37). Steroid side effects such as mood changes, metabolic derailment, sleep disorders, and myopathy add to the symptoms of advanced cancer and can further impair the quality of life (9). Due to steroids’ adverse complications, they often do not provide long-term efficacy. In addition, steroids combined with mannitol have poor efficacy in refractory cerebral edema, with a control rate of approximately 30% (38, 39). Bevacizumab has been reported to improve steroid-resistant cerebral edema. A previous study reported that bevacizumab treatment resolved edema in 82% of patients (5). In our study, the edema control rate was similar at 84.72%.

This study is the first to assess differences in the therapeutic efficacy of bevacizumab on refractory brain edema caused by brain metastasis from different tissues of origin: lung and colon. These findings may have important clinical significance for the treatment of these patients. Previous studies have shown that bevacizumab treats brain edema by blocking the binding of VEGF-A to its receptor (40–43). Zustovich reported on 18 patients with peritumoral cerebral edema treated with bevacizumab. The objective control rate was 100%, and the effective rate was 60% (44).

In this study, patients were reexamined by MRI 4 weeks after treatment. A total of 61 patients (84.72%) achieved edema control after a single bevacizumab treatment. We found that brain edema was controlled in 40 patients (93.02%) in the lung cancer group and 21 patients (72.41%) in the colon cancer group (P=0.023). This observation confirms that bevacizumab has differential efficacy in refractory cerebral edema caused by brain metastases from different organs. Refractory brain edema from colon cancer brain metastases may require higher doses of bevacizumab. In addition, 22 patients (88.00%) in the radiotherapy group achieved edema control compared to 39 (82.98%) in the non-radiotherapy group (P=0.573). We found no significant difference in the edema control rate between the radiotherapy and non-radiotherapy groups. This may suggest that bevacizumab can effectively alleviate radiation-induced brain edema.

Patients were followed up for 1 month. We only examined changes in refractory brain edema after a single bevacizumab treatment. In our study, the average edema volume before treatment was 201,708.97 ± 61,426.04 mm^3^ which has decreased to 116,947.01 ± 43,879.16 mm^3^ after treatment. These results showed that bevacizumab reduces the volume of refractory brain edema. Even if edema control is achieved after a single treatment, some patients retain a large volume of peritumoral edema. These patients continued bevacizumab treatment 4 weeks after the first treatment. Due to the short follow-up time, we could only observe the short-term effect of bevacizumab on refractory cerebral edema. Therefore, the long-term efficacy of bevacizumab after withdrawal is unclear. However, a previous study has reported that bevacizumab was effective in relapsed refractory cerebral edema (45). Furthermore, considering the short survival time of patients with brain metastases, we believe that the role of bevacizumab is worthy of recognition.

When bevacizumab was ≥0.3 mg/kg, free VEGF in the serum could not be detected (46). The currently recommended therapeutic dose of bevacizumab is 5–10 mg/kg (11–14). Although there is no evidence that adverse drug reactions are related to the dose, we still choose safer therapeutic doses. In our study, the treatment dose of bevacizumab was 5 mg/kg. Nine patients developed hypertension after a single bevacizumab treatment and returned to normal after nifedipine treatment. Platelet levels decreased in two patients and returned to normal without treatment. Some studies have shown that bevacizumab-induced hypertension significantly predicts progression-free survival and overall survival in patients with metastatic colorectal cancer, whereas its prediction for the objective response rate was non-significant (47, 48). Previous studies have reported that hypertension may be an indicator of positive antitumor effects, may predict the efficacy of antiangiogenic therapy, and could be associated with a favorable tumor prognosis (49). In our study, the edema volume was reduced by 98,237.81 ± 32,134.05 mm^3^ in nine patients with new-onset hypertension, while the edema volume in the others was reduced by 83,351.25 ± 47,735.24 mm^3^ (P<0.05). The edema control rate in the hypertension group was 88.89%, while the edema control rate in patients without new-onset hypertension was 84.13% (P>0.05). Compared to non-hypertensive patients, hypertensive patients exhibited a more significant reduction in the edema volume, but there were no significant differences in the edema control rate between the two groups. Hypertension may be used to predict the efficacy of bevacizumab in refractory cerebral edema, but more research is needed to demonstrate this.

Despite these findings, our study has some limitations. First, this was a single-center study. If we can conduct further multicenter research, the results will be more representative. Second, radiotherapy during follow-up may have an impact on the edema volume and edema index. Our study found for the first time that bevacizumab has a differential efficacy of refractory brain edema caused by brain metastases from primary lung and colon cancers. However, the reasons for the differences in efficacy need to be further studied. Finally, only 72 patients were included in this study. All patients received only a single dose of bevacizumab with a short follow-up period. Increasing the follow-up time and bevacizumab dose allows for a more precise assessment of bevacizumab efficacy.

## Conclusion

This study suggests that bevacizumab may reduce refractory brain edema, and there is a significant difference in the efficacy of bevacizumab on refractory brain edema caused by the brain metastasis of lung cancer and colon cancer. A total of 11 patients experienced mild adverse reactions and quickly returned to normal. Therefore, bevacizumab is a safe and effective treatment option for refractory brain edema.

## Data availability statement

The original contributions presented in the study are included in the article/supplementary material. Further inquiries can be directed to the corresponding author.

## Ethics statement

The studies involving human participants were reviewed and approved by the Academic and Ethical Committee of the First Affiliated Hospital of Jinan University. The patients/participants provided their written informed consent to participate in this study.

## Author contributions

XB and MZ participated in the design of this study and collected important background information. XB completed related literature retrieval, data acquisition and data analysis. XB drafted the manuscript, MZ completed the revision of the manuscript. All the authors read and approved the final manuscript. MZ are responsible for the final manuscript. The authors declare that there are no conflicts of interest. All authors contributed to the article and approved the submitted version.

## Acknowledgments

The authors are grateful to all patients included in this study for their support.

## Conflict of interest

The authors declare that the research was conducted in the absence of any commercial or financial relationships that could be construed as a potential conflict of interest.

## Publisher’s note

All claims expressed in this article are solely those of the authors and do not necessarily represent those of their affiliated organizations, or those of the publisher, the editors and the reviewers. Any product that may be evaluated in this article, or claim that may be made by its manufacturer, is not guaranteed or endorsed by the publisher.
